# Prime incision: A minimally invasive approach to breast cancer surgical treatment—A 2 cohort retrospective comparison with conventional breast conserving surgery

**DOI:** 10.1371/journal.pone.0191056

**Published:** 2018-01-18

**Authors:** Silvio Eduardo Bromberg, Patricia Rodrigues Alves de Figueiredo Moraes, Felipe Ades

**Affiliations:** 1 Centro de Oncologia, Hospital Israelita Albert Einstein, São Paulo, Brazil; 2 Universidade Federal do Estado de São Paulo, São Paulo, Brazil; 3 Centro de Referência da Saúde da Mulher, Hospital Perola Byington, São Paulo, Brazil; University of North Carolina at Chapel Hill School of Medicine, UNITED STATES

## Abstract

The prime incision technique is an oncoplastic surgery aimed to remove both the breast tumor and the sentinel lymph node through one incision, thus providing better aesthetic results than the conventional breast conservative two incision technique. We retrospectively evaluated 2 cohorts of 60 consecutive breast cancer patients operated by either conventional breast conservative surgery (N = 26) or one incision surgery (N = 34). There were no recurrence or death events observed in any group. No difference was seen regarding the incidence of surgical complications. In the prime incision group the breast volume removed was significantly lower than in the conventional surgery group as well as was the surgical time and the number of dissected lymph nodes. Aesthetical results were better in the one incision group. Further prospective studies are needed to validate the one incision technique as a surgical option for selected early stage breast cancer patients.

## Introduction

Breast conserving surgery was developed to avoid mastectomy and has become the standard of care in early stage breast cancer. Breast conserving treatment aims to achieve an acceptable cosmetic result while achieving local disease control [1]. Several randomized trials have shown that mastectomy with axillary dissection is equivalent to breast conserving surgery with axillary dissection and whole breast irradiation, as primary breast treatment for most women with stage I and II breast cancer [2].

In cases in which the proportion between the breast’s and tumor’s volume allow an oncologic resection, the conservative option is less invasive, faster and more aesthetic. Exceptions to this approach would be contraindications such as previous radiation therapy to the breast, active connective tissue disease involving skin, the majority of tumors greater than 5 cm without neoadjuvant chemotherapy, and focally positive pathologic margins [1–3].

Patient concerns with aesthetics have led to the development of oncoplastic surgical approaches. It has been demonstrated that the aesthetic success in breast cancer surgical treatment leads to improved sexual and social recovery [4,5]. In patients that have no desire or no need for associated mammoplasty, minimally invasive treatments allow the maintenance of the breast pre-surgical appearance. The prime incision technique is an oncoplastic surgery aimed to remove both the breast tumor and the sentinel lymph node through one incision, thus providing better aesthetic results than the conventional breast conservative two incision technique. However this procedure is more difficult, since visualization and the resection space are limited, demanding greater experience from the surgeon.

This surgical option was offered to early breast cancer patients that desired a good cosmetic result and in which the prime incision surgery was considered safe, ie. tumors measuring no more than 25 mm and clinically negative axillary lymph nodes. We have previously described the safety and efficacy of the prime incision technique [6]. In this study we present the results of two retrospective cohorts of early stage breast cancer patients treated with breast-conserving surgery. The first cohort was treated with the prime incision approach and the second cohort was treated by the conventional breast conservative technique, which is performed with double incision (one for the tumor resection and the other for the axillary dissection).

## Methods

The objective of the study was to compare the oncological safety and aesthetical results between the prime incision technique and the conventional breast conserving surgery. We compared the mammary volume removed; surgical time; number of dissected lymph nodes; surgical complications such as seroma, infection, and dehiscence of the surgical wound; and deformities, retractions, and subsequent esthetic sequelae.

We identified the patient´s charts of 60 consecutive early breast cancer patients treated with breast conserving surgery between January 2013 and December 2015. After the initial description of the prime technique this became of preferred surgical approach [6]. We included all consecutive patients operated at our hospital before and after the adoption of the prime technique. Patients were documented with clinical and imaging exams and had breast cancer confirmation previous to surgery with core needle biopsies [7,8]. The selected patients had a single-lesion invasive breast cancer, measuring no more than 25 mm with clinically negative axillary lymph nodes. Patients were operated by same surgical team, composed by one surgeon and one assistant.

### Surgical technique

Patients were injected with 0.2 mL of dextran marked with radiotracer (technetium—Tc99m) with 0.4 mCi activity around the tumor and under the areola approximately 4 hours before the surgical procedure. Non-palpable sentinel lymph nodes were identified through intra-operative tracking with Gamma-Probe [8].

**Fig 1:**
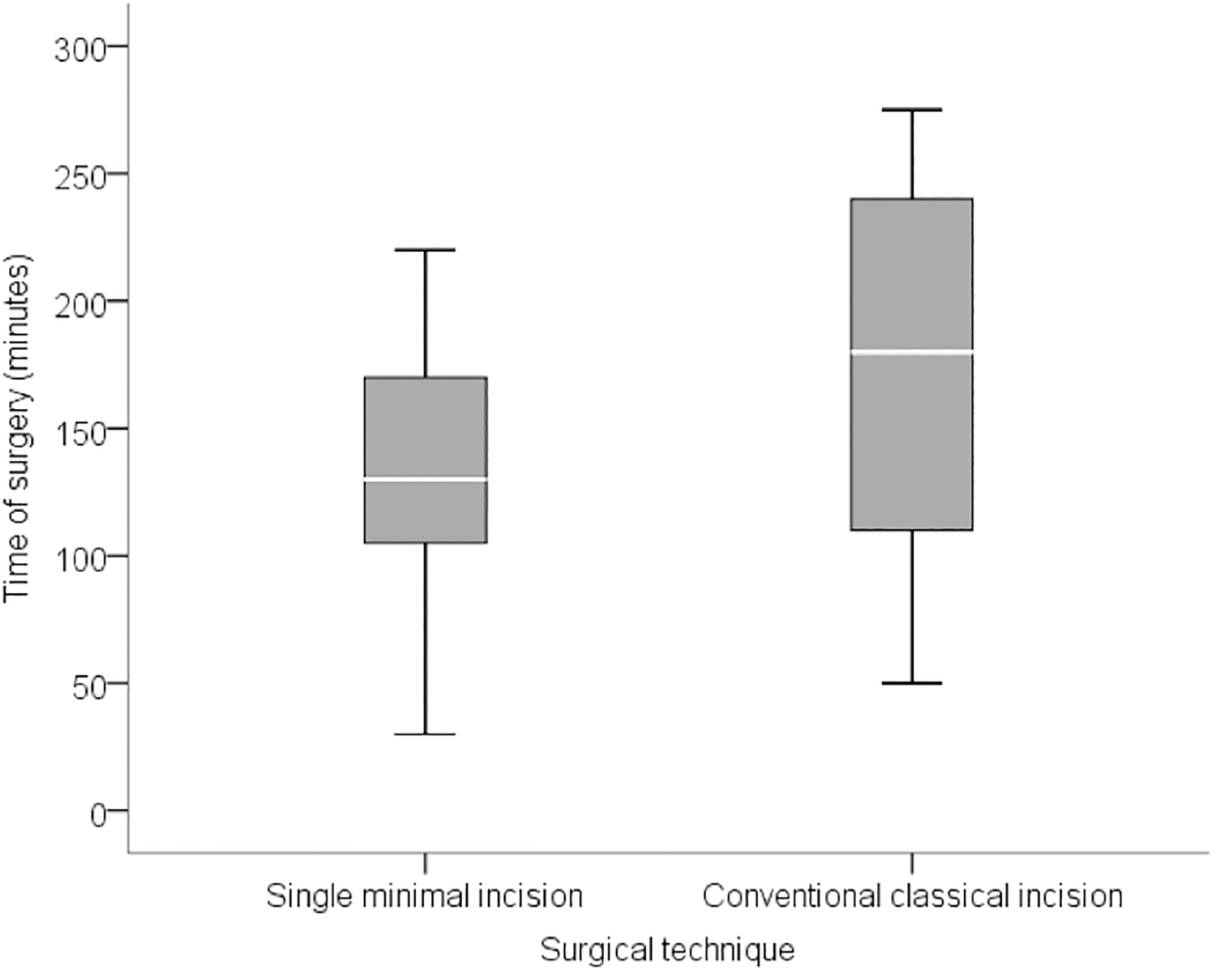
Median surgical time (in minutes).

**Fig 2:**
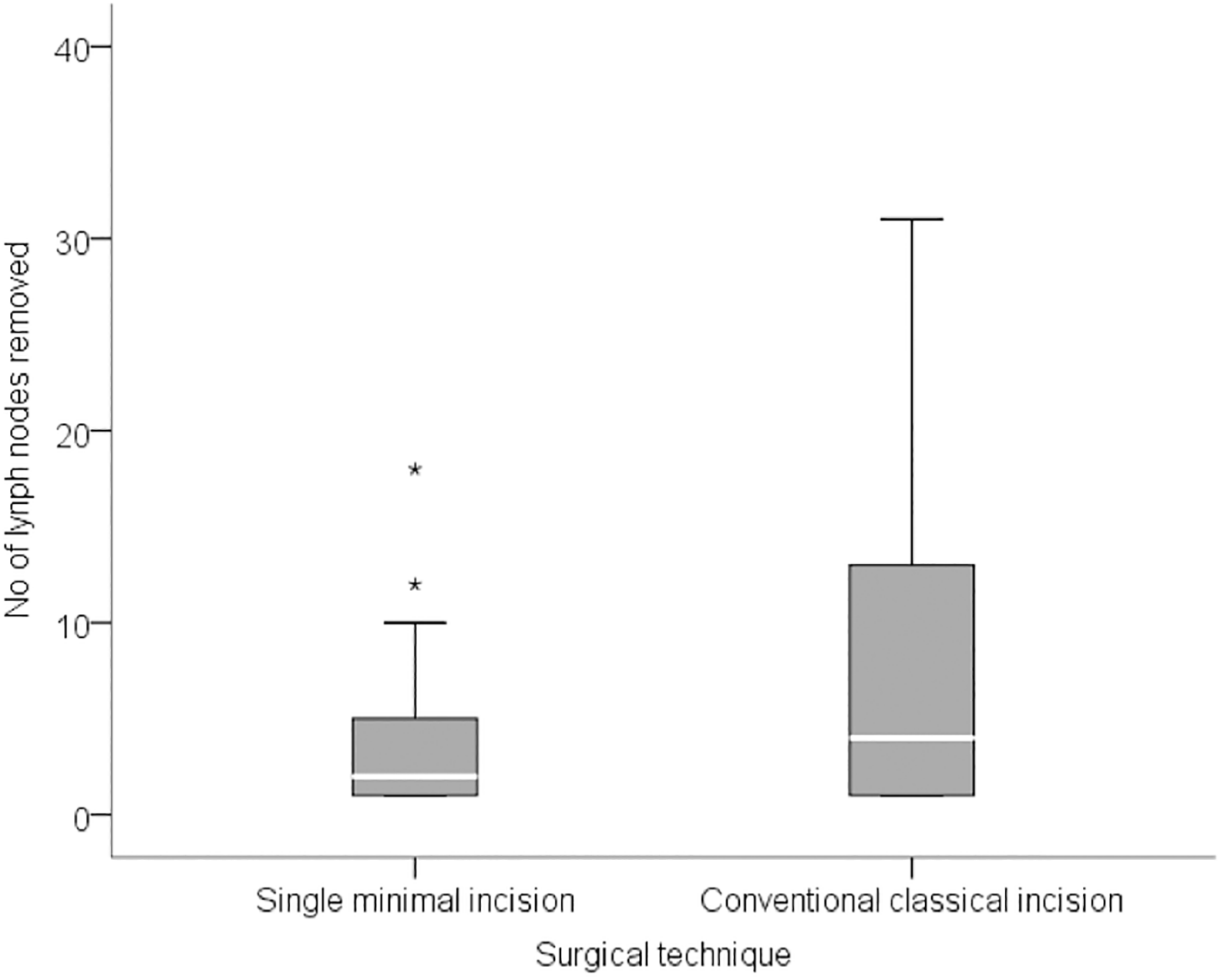
Number of dissected lymph nodes.

In the prime incision group, we used the wound retractor (XS model), and dissections were performed using a mono-polar electric scalpel [9]. During the surgery, we used either a single periareolar incision involving half of the areola circumference, or a single incision in the mammary sulcus of 4 to 7 centimeters in length, in cases for whom the areola had less than 3 centimeters of diameter or when the patient previously had such incision (Figs 1 and 2).

After the incision, a cutaneous pouch was made in the edge of the surgical wound so we could insert the retractor. With the protection and maintenance of the opening of the incision by the wound retractor, the exposure of the surgical area was improved in comparison to the usual retractors [9].

Through the prime incision, over the wound retractor, we used long bright-lighted retractors and a smoke aspirator to improve visibility. We continued the dissection of the cutaneous pouch through the superficial fascia, peeling off the glandular tissue until the tumor area could be accessed. We then dissected the lesion and adjacent tissue, separated it from the deep fascia, and removed it.

**Fig 3:**
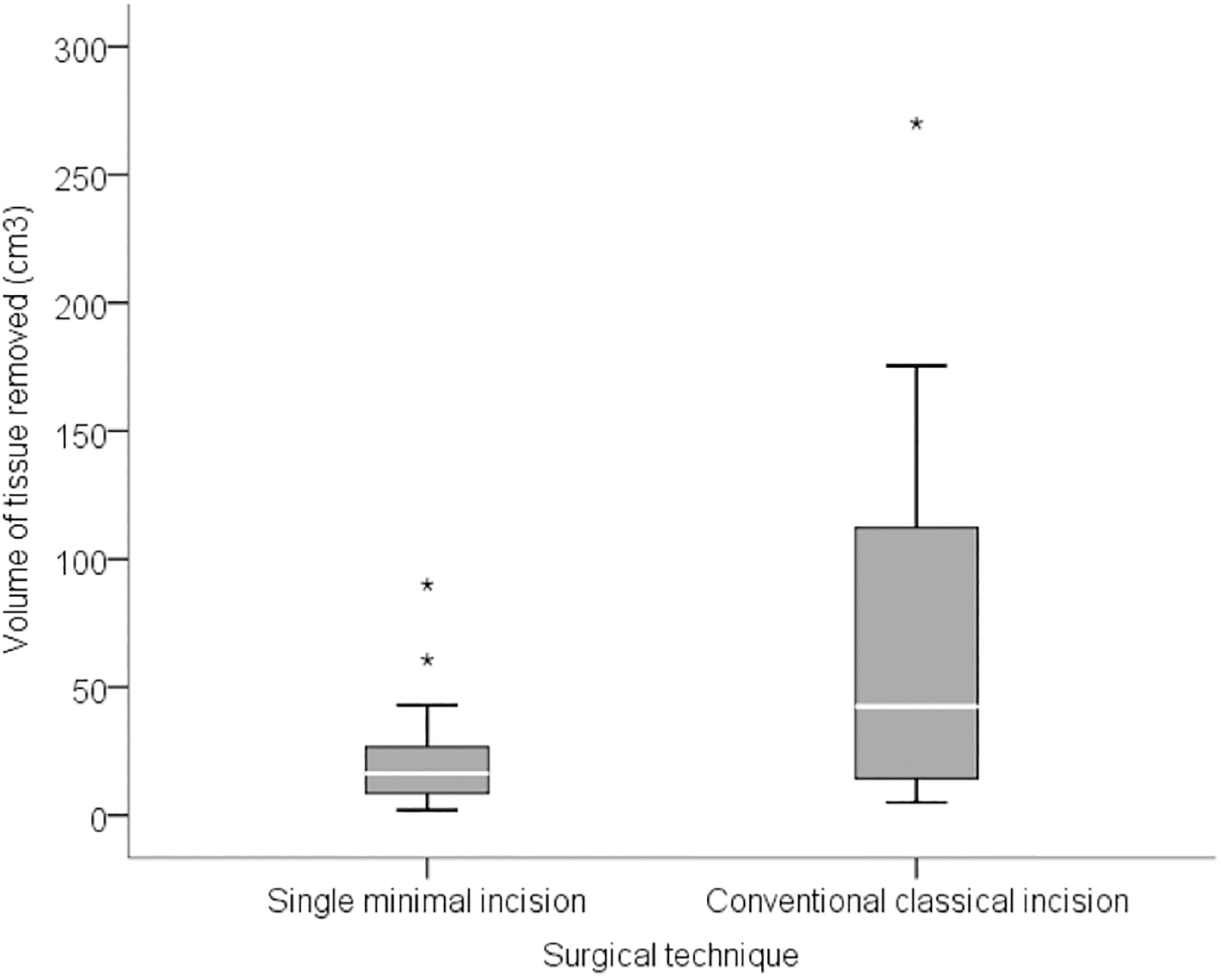
Volume of tissue removed (in cm3).

Following the lesion removal with assurance of proper borders we performed the shaving of the margins after the segmental dissection. This was made to ensure that there were no compromised margins after the surgery [10]. After we detached the mammary gland from the deep fascia, we could reach the axillary region for identification and removal of sentinel lymph nodes. If necessary, other axillary lymph nodes were dissected and removed. One case underwent an exclusively axillary approach after the incision, the retractor was inserted, and the dissection proceeded as described previously (Fig 3).

The conventional breast conservative surgery group underwent surgery using one incision for tumor removal, either above the tumor, or in the periareolar region, and a second 3 cm incision in the axillary area for lymph node exploration. The wound retractor was not used in these patients.

Both groups received a vacuum aspirator drain with a single skin exit. Resected material was intraoperatively analyzed by frozen section for margins and sentinel lymph node(s) status. After surgery, specimens were sent for regular anatomopathological evaluation.

Patients were followed weekly in the first 30 days after the surgery and at every 3 months for the following two years.

### Data analyses

The descriptions were carried out using absolute frequencies and percentages in the case of categorical variables and means and standard deviations or medians and quartiles, as well as minimum and maximum values, in the case of quantitative variables. To evaluate the homogeneity of the groups, we applied Student’s t-test in the comparison of average age and the Fisher’s exact test for the comparison of distribution of the disease staging. Generalized linear models in multiple approaches were adjusted to assess the association between outcome variables and the surgical technique, controlling for disease staging. Analyses were performed using SPSS for Windows version 17.0 (SPSS Inc., Chicago, IL, USA) and significance was set at p<0.05.

Patients provided written informed consent to have their medical record data used in research. The study was performed with approval of the Ethical Committee from Hospital Israelita Albert Einstein (SGPP 2662). The individuals in this manuscript have given written informed consent to publish these case details, including the use of photographs in medical publications.

## Results

From the 60 cases identified in our patient´s records, 34 were operated through the prime incision technique and 26 through the conventional technique. Demographic and surgical data are presented in Table 1.

**Table 1:** Demographics and surgical results.

	Prime incision (n = 34)	Conventional surgery (n = 26)	P value
**Age**			0.241
**Median**	53.9 (11.4)	57.4 (11.3)	
**Range**	33–76	34–85	
			
**Disease stage**			0.482
**I**	30 (88%)	21 (81%)	
**II**	4 (12%)	5 (19%)	
			
**Disease location**			--
**Infero lateral**	9 (26%)	4 (15%)	
**Intero medial**	4 (12%)	1 (4%)	
**Supero lateral**	16 (47%)	11 (42%)	
**Supero medial**	4 (12%)	7 (27%)	
**Retro areolar**	1 (3%)	3 (12%)	
			
**Incision**			>0.99
**Axilla**	1 (3.1)	0 (0.0)	
**Periareolar**	21 (65.6)	17 (65.4)	
**Sulcus**	10 (31.3)	9 (34.6)	
			
**Breast dissected volume (mm3)**			<0.001
**Median (range)**	16.3 (2–90)	42.4 (5–270)	
			
**Positive lymph nodes**			--
**0**	28 (90.3%)	16 (61.5%)	
**1**	2 (6.5%)	5 (19.2%)	
**3**	1 (3.2%)	0 (0.0%)	
**9**	0 (0.0%)	3 (11.5%)	
**10**	0 (0.0%)	1 (3.8%)	
**16**	0 (0.0%)	1 (3.8%)	
			
**Dissected lymph nodes**			<0.001
**median (IIQ)**	2 (1–5)	4 (1–13)	
**Range**	1–18	1–31	
			
**Surgical time (min)**			0,010
**Median (range)**	130 (30–220)	180 (50–275)	

The mean age was 53.9 years in the prime incision technique group and 57.4 years in the conventional technique group (p = 0.241). Most patients were stage 1 (88.2% in prime incision group and 80.8% in conventional group (p = 0.482)).

The volume of tissue removed in the prime incision group ranged from 2 cm^3^ to 90 cm^3^, with a median volume of 16.3 cm^3^ (IQR: 8.5 to 26.7 cm^3^) while in the conventional group it ranged from 5cm^3^ to 270 cm^3^, with a median volume of 42.4 mm^3^ (IQR: 14.4 to 112.2 cm^3^). The number of lymph nodes removed ranged from 1 to 18 (median 2, IQR: 1 to 5) in the prime incision group, and from 1 to 31 (median 4, IQR: 1 to 13) in the conventional group. The surgical time in the prime incision group ranged from 0.5 to 3.7 hours with a median time of 2.2 hours (IQR: 1.8 to 2.8 hours). In the conventional technique group surgical time ranged between 0.8 and 4.6 hours, with a median of 3.0 hours (IQR: 1.8 to 4.0 hours). There was no correlation between surgical time and disease staging (p = 0.847). Data are summarized in Figs 1–3.

**Fig 4:**
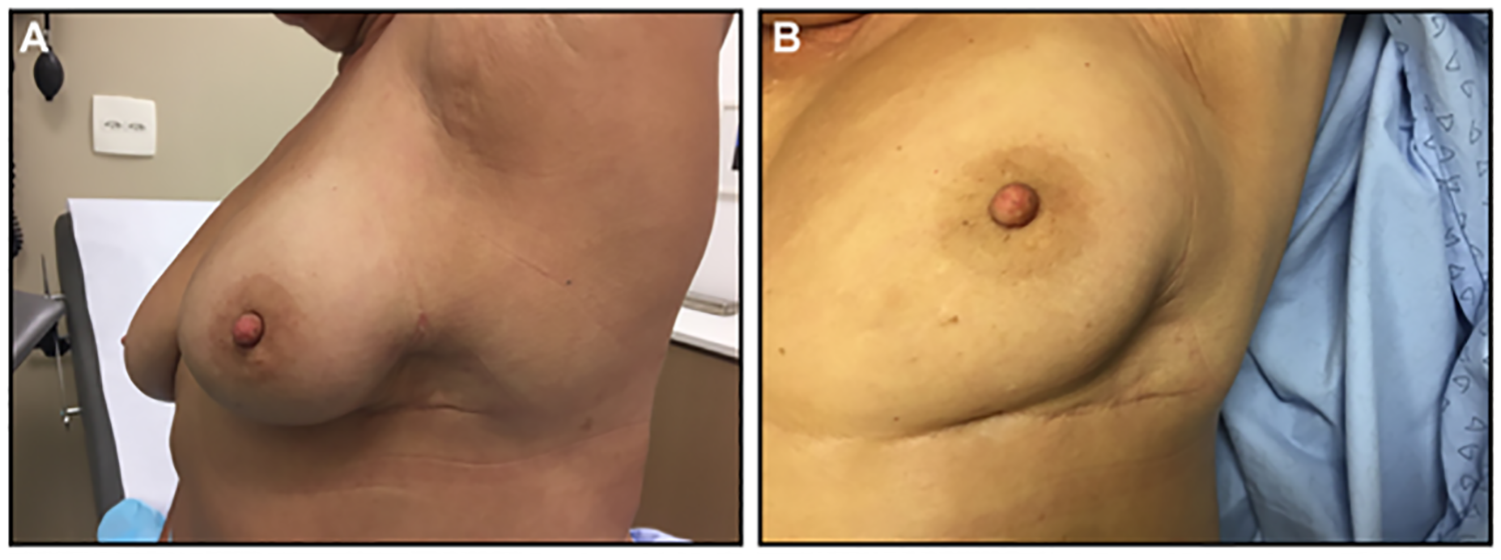
Postoperative retraction.

Complications in the prime incision group included two cases of pruritic eczematous lesions in the operated breast, successfully treated with corticoid-base cream. Another complication observed was one case in which the tumor was in the inferolateral quadrant, which presented a skin retraction when the patient lifted her arm (Fig 4). No case of seroma persisting for over 10 days, dehiscence, or infection was observed in either group. The median post-operative follow-up was 7.5 months, ranging from 1 to 16 months.

At the time of this analysis there were no recurrence or death events observed in any of the groups.

## Discussion

Breast surgery is the main treatment for breast cancer. The Halsted mastectomy was the first surgical technique able to, scientifically and systematically, cure a fraction of breast cancer patients. Nevertheless, this technique lead to significant comorbidities and unsatisfactory aesthetic results [11].

The subsequent development of radiotherapy and systemic treatments has allowed further development of breast cancer surgery. The work of Bernard Fisher and Umberto Veronesi has showed similar results of partial mastectomy and radiotherapy in relation to total mastectomy in terms of disease control, with improvements in breast cosmetics [12,13]. After the results of the pivotal studies NSABP-B06 and the Milan I, II and III trials, breast conservative surgery followed by radiotherapy had become the standard treatment for early stage breast cancer [14,15].

Improvements in the axillary surgery approach, with the sentinel lymph node biopsy, allowed de-escalation of surgical comorbidities, maintaining high accuracy of metastatic spread to this location. Both techniques used nowadays, the patent blue dye injection and the technetium gamma ray detection, are considered appropriated for this purpose [16,17].

In a previous study we have described the prime incision surgical technique, a technique that requires additional surgical training, as the incision is narrower, and dissections need to be performed with long devices, besides the issues related to local lighting. Nevertheless, it is able to reduce the size of resections with a single incision, which allows better cosmetic results [6]. In the present paper, we present the results of cohort study comparing the prime incision technique with the conventional breast conserving surgery technique.

Our results have shown, indeed, smaller mammary volume dissected in the prime incision group, maintaining adequate disease control. As we do perform routinely margins shaving after the segmental dissection, we did not observe any case of compromised margins and no subsequently re operations were needed [10]. No local recurrences were observed in neither group.

The number of dissected lymph nodes was also smaller in the prime incision group. The sentinel lymph node was identified in all cases and, in no case, further axillary dissection was needed. This result shows that the prime incision technique does not hampers the ability to find the sentinel lymph node, also reflecting the strict patient selection for this technique. Surgical time was also shorter in the prime incision technique. The shorter time was due to saving time in multiple surgical procedures as the absence of a second approach to the axilla, a shorter time for drain positioning, breast reconstruction and skin stitching. In both groups, there were no short-term or late complications postoperatively (seroma, anatomical deformities, fibrosis or infection).

Although the prime incision technique reduces the surgical time, the number of dissected lymph nodes and the dissected mammary volume, it demands additional surgical learning and skills from the surgeon, as it requires a narrower incision with limited view of the surgical resected area. There are also contraindications for this technique, as the absence of adequate surgical equipment, the lack of appropriately trained staff, in addition to the usual contraindications for breast conservative surgery.

The fact that the breast and axillary approaches are done through a single incision raises concerns about seroma contamination. In theory, in the two-incision technique, a separate approach to the axilla would avoid possible metastatic spread from cancerous cells from the breast to the axilla, though the single seroma cavity of the prime technique. The medical literature on this topic is scarse, two studies have addressed this issue. In a series of 142 patients it was observed, indeed, a higher seroma contamination and this was associated with the menstrual cycle and with a higher chance of locoregional recurrence. Nevertheless, this study has several limitations that humpers the ability to extrapolate its data to today’s breast surgery approaches. The study dates from the 80’s, when the adjuvant treatment with chemotherapy and radiotherapy was not widespread and not as effective as the current treatments [18].

In a second, more recent study, 68 patients were analyzed for the presence of cancerous cells, of these 4 patients were positive. Three of these patients had had complete axillary dissection due to the presence of clinically positive lymph nodes and one was dissected because of a positive sentinel lymph node. This reflects a higher tumoral burden in the axilla and a wider lymphatic vessels damage, both situations that could impact seroma contamination [19].

In our study only 5 patients had more than 9 lymph nodes dissected and all of these patients were in the conventional two incision group. Patients, in both groups, received adjuvant systemic treatment, as indicated by their staging and molecular profile, and received adjuvant radiotherapy, as preconized by international guidelines, including tangential fields targeting the axillary Berg’s level 1.

In conclusion, the prime incision technique was shown to be similar to the conventional breast conserving surgery in terms of oncologic outcomes, thus providing better cosmetic result. We foresee that this technique has the potential to be associated, in the future, to other surgical approaches as video assisted surgery. Randomized prospective validation of the prime incision technique is needed to establish this technique in the surgical armamentarium for the treatment of selected early breast cancer patients.

## Supporting information

S1 Table Patients’ staging, disease characteristics and surgical time. (DOCX)
