# Correction: Prime incision: A minimally invasive approach to breast cancer surgical treatment—A 2 cohort retrospective comparison with conventional breast conserving surgery

**DOI:** 10.1371/journal.pone.0195130

**Published:** 2018-03-26

**Authors:** Silvio Eduardo Bromberg, Patricia Rodrigues Alves de Figueiredo Moraes, Felipe Ades

The second sentence of the second paragraph under the “Surgical technique” heading of the Methods section is incorrect. The correct sentence is: During the surgery, we used either a single periareolar incision involving half of the areola circumference, or a single incision in the mammary sulcus of 4 to 7 centimeters in length, in cases for whom the areola had less than 3 centimeters of diameter or when the patient previously had such incision (Figs 5 and 6).

**Fig 5:**
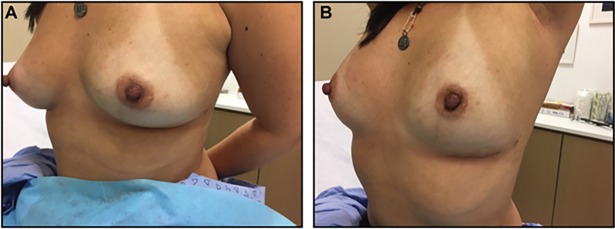
Inframammary one incision.

**Fig 6:**
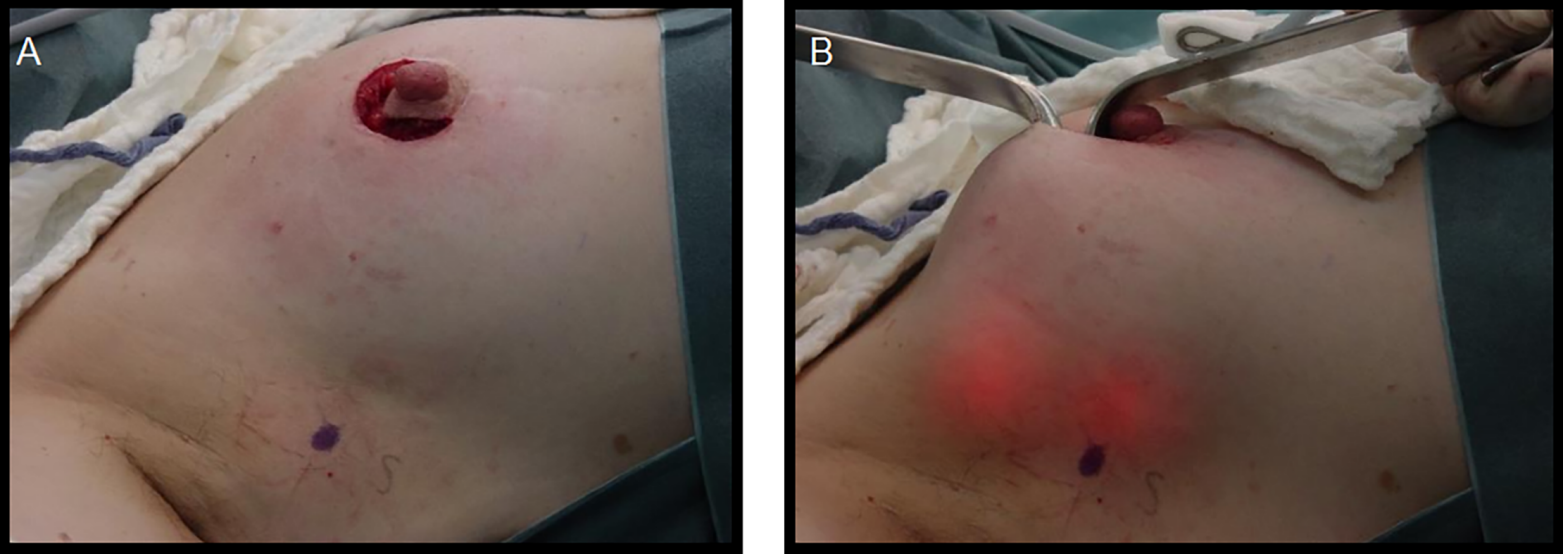
Periareolar one incision.

The last sentence of the fifth paragraph under the “Surgical technique” heading of the Methods section is incorrect. The correct sentence is: One case underwent an exclusively axillary approach after the incision, the retractor was inserted, and the dissection proceeded as described previously (Fig 7).

**Fig 7:**
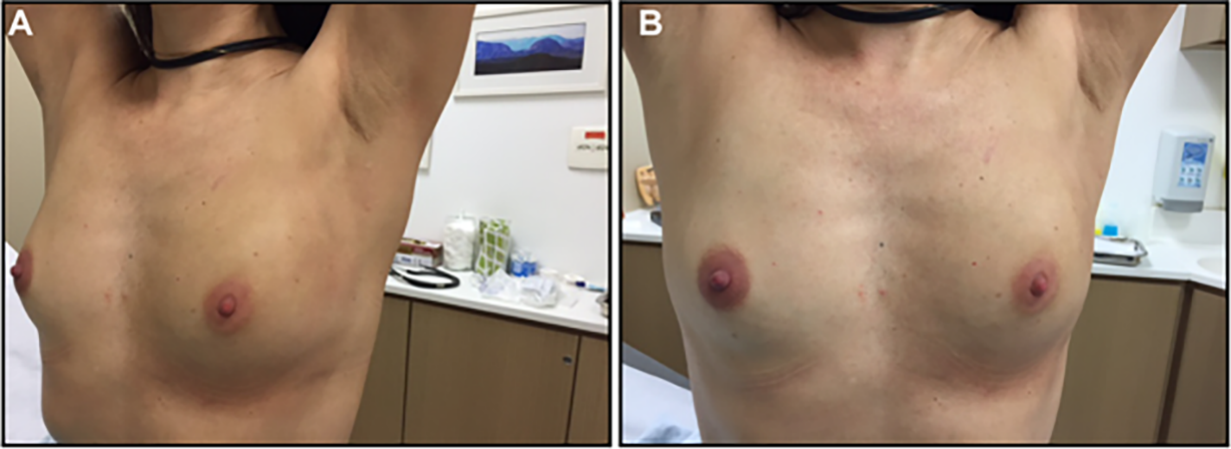
Axillary one incision.

Figs 5, 6 and 7 are omitted. Please see Figs 5, 6 and 7 below.
