# Supplementary material for: Prime incision: A minimally invasive approach to breast cancer surgical treatment—A 2 cohort retrospective comparison with conventional breast conserving surgery
Source: PLoS One. 2018 Jan 18;13(1):e0191056. doi: 10.1371/journal.pone.0191056 (PMC5773166; doi:10.1371/journal.pone.0191056)
Supplement: S1 Table — (DOCX) [file pone.0191056.s001.docx]

| One incision | Tumoral  Size - mm | T stage | Stage | Localization | Volume breast tissue- mm3 | Surgical time |
| --- | --- | --- | --- | --- | --- | --- |
| 1 | 12 | T1c | I | QSL | 9 | 13:40-15:55 |
| 2 | 10 | T1b | I | QSL | 9 | 16:30-18:15 |
| 3 | 11 | T1c | I | QQLL | 12 | 16:35-18:45 |
| 4 | 16 | T1c | I | QSL | 15,7 | 14:15-16:00 |
| 5 | 9 | T1b | I | QIM | 20,9 | 11:00-12:25 |
| 6 | 18 | T1c | I | QQLL | 32,6 | 11:45-13:45 |
| 7 | 23 | T2 | II | QQLL | 60,6 | 15:40-18:30 |
| 8 | 14 | T1c | I | QSL | 32,6 | 12:55-15:05 |
| 9 | 9 | T1b | I | QIL | 17,5 | 14:50-16:45 |
| 10 | 4 | T1a | I | QSM | 2 | 08:10-11:20 |
| 11 | 5 | T1a | I | QSL | 2 | 14:00-17:00 |
| 12 | 6 | T1b | I | QQLL | 8 | 16:44-18:35 |
| 13 | 23 | T2 | II | QSL | 26,3 | 16:35-16:30 |
| 14 | 11 | T1c | I | QQLL | 90 | 14:45-15:40 |
| 15 | 12 | T1c | I | QSL | 23,9 | 13:05-16:35 |
| 16 | 20 | T1c | I | INTERPEITORAL | 13,7 | 13:50-15:40 |
| 17 | 13 | T1c | I | QQLL | 16,8 | Missing |
| 18 | 11 | T1c | I | QQMM | 22,7 | Missing |
| 19 | 16 | T1c | I | QIL | Missing | Missing |
| 20 | 9 | T1b | I | QQLL | 4 | 16:00-17:35 |
| 21 | 10 | T1b | I | QIM | 13,7 | 12:50-14:20 |
| 22 | 11 | T1c | I | QSM | 6,6 | Missing |
| 23 | 8 | T1b | I | QQSS | 6,6 | 12:50-15:45 |
| 24 | 9 | T1b | I | RRA | 19 | 12:45-14:30 |
| 25 | 8 | T1b | I | QIL | 8 | 16:30-19:10 |
| 26 | 11 | T1c | I | QSL | 43 | 17:15-19:30 |
| 27 | 8 | T1b | I | QIL | 5 | 16:20-18:00 |
| 28 | 10 | T1b | I | QQII | 12,6 | 14:00-16:50 |
| 29 | 14 | T1c | I | QSL | 40,25 | 16:50-17:20 |
| 30 | 20 | T1c | I | QQSS | 13,8 | 14:15-17:05 |
| 31 | 14 | T1c | I | QSM | 13 | 15:00-17:20 |
| 32 | 12 | T1c | I | QSL | 16,8 | 14:00-15:45 |
| 33 | 10 | T1b | I | QQLL | 39,9 | 15:40-19:20 |
| 34 | 12 | T1c | I | QIL | 27 | 14:20-17:00 |

| CONVENCIONAL SURGERY | Tumoral Size - mm | T stage | Stage | Localization | Volume breast tissue  CC3 | Surgical time |
| --- | --- | --- | --- | --- | --- | --- |
| 1 | 7 | T1b | I | QSL | 10,84 | 14:00-17:10 |
| 2 | 11 | T1c | I | QQMM | 14,4 | 1445:18:45 |
| 3 | 18 | T1c | I | QQMM | 106,14 | 14:20-18:00 |
| 4 | 14 | T1c | I | QQLL | 48,16 | 14:00-17:00 |
| 5 | 5 | T1a | I | QQSS | 18 | 14:30-16:00 |
| 6 | 7 | T1b | I | QSL | 36,7 | Missing |
| 7 | 10 | T1b | I | QQII/QIL | 12 | 14:30-15:20 |
| 8 | 19 | T1c | I | QSM | 114,5 | 10:45-15:10 |
| 9 | 10 | T1b | I | RRA | 15,9 | 16:50-18:40 |
| 10 | 22 | T2 | II | RRA | 165,7 | 07:15-12:50 |
| 11 | 15 | T1c | I | QSM | 50,4 | 16:50-22:35 |
| 12 | 7 | T1b | I | QQII | 270 | 8:20-12:55 |
| 13 | 6 | T1b | I | QSM | 56,2 | 15:10-00:55 |
| 14 | 16 | T1c | I | QSM | 112,2 | 11:55-16:20 |
| 15 | 23 | T2 | II | QSL | 145,8 | Missing |
| 16 | 22 | T2 | II | QSL | 50,9 | 14:25-17:40 |
| 17 | 40 | T2 | II | QQLL | 175,5 | 11:05-16:15 |
| 18 | 12 | T1c | I | QQSS | 30,4 | 13:50-16:45 |
| 19 | 12 | T1c | I | QQSS | 82,8 | Missing |
| 20 | 8 | T1b | I | QQII | 33,2 | 12:15-13:40 |
| 21 | 35 | T2 | II | RRA | 162,4 | Missing |
| 22 | 8 | T1b | I | QQLL | 11 | 13:50-16:50 |
| 23 | 3 | T1a | I | QSL | 7 | 15:50-17:35 |
| 24 | 6 | T1b | I | QQLL | 5 | 08:00-12:10 |
| 25 | 25 | T2 | II | QSM | 20 | 08:00-17:10 |
| 26 | 26 | T2 | II | QSM | 9 | 15:35-18:00 |
